# Recent Advances in Phthalocyanine-Based Hybrid Composites for Electrochemical Biosensors

**DOI:** 10.3390/mi15091061

**Published:** 2024-08-23

**Authors:** Keshavananda Prabhu Channabasavana Hundi Puttaningaiah, Jaehyun Hur

**Affiliations:** Department of Chemical, Biological, and Battery Engineering, Gachon University, Seongnam-si 13120, Gyeonggi-do, Republic of Korea; keshavmgm@gmail.com

**Keywords:** biosensors, phthalocyanine, nanoparticles (NPs), carbon nanomaterials, hybrid materials, biomolecule detection

## Abstract

Biosensors are smart devices that convert biochemical responses to electrical signals. Designing biosensor devices with high sensitivity and selectivity is of great interest because of their wide range of functional operations. However, the major obstacles in the practical application of biosensors are their binding affinity toward biomolecules and the conversion and amplification of the interaction to various signals such as electrical, optical, gravimetric, and electrochemical signals. Additionally, the enhancement of sensitivity, limit of detection, time of response, reproducibility, and stability are considerable challenges when designing an efficient biosensor. In this regard, hybrid composites have high sensitivity, selectivity, thermal stability, and tunable electrical conductivities. The integration of phthalocyanines (Pcs) with conductive materials such as carbon nanomaterials or metal nanoparticles (MNPs) improves the electrochemical response, signal amplification, and stability of biosensors. This review explores recent advancements in hybrid Pcs for biomolecule detection. Herein, we discuss the synthetic strategies, material properties, working mechanisms, and integration methods for designing electrochemical biosensors. Finally, the challenges and future directions of hybrid Pc composites for biosensor applications are discussed.

## 1. Introduction

**Figure 1:**
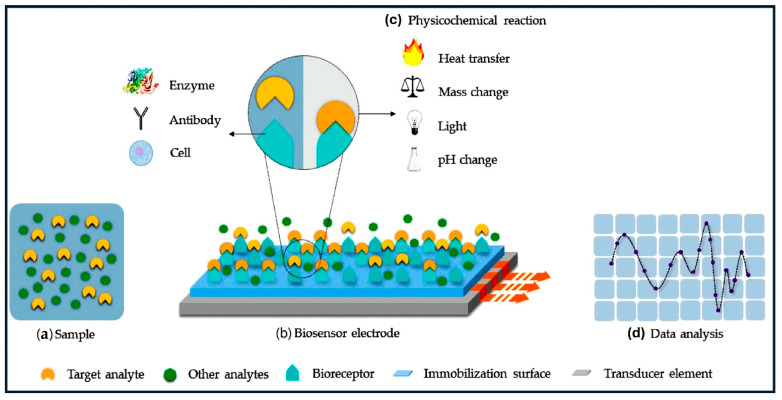
The schematic of a biosensor device, where the target analyte (**a**) interacts with the bioreceptor (**b**) immobilized on an electrode surface. This interaction triggers a physicochemical reaction (**c**) that the transducer element converts into a measurable signal. This signal is then analyzed (**d**) to detect and quantify the target analyte [10].

The pervasive conveniences of modern life are a testament to the remarkable advancements in science and technology. We consistently rely on various devices such as computers, phones, refrigerators, air conditioners, and smoke detectors for seamless interaction with the physical world [1]. Most of these devices would not operate without sensors, which act as our electronic eyes and ears [2]. Electronic devices detect changes in physical or chemical properties, such as pressure, temperature, humidity, motion, and light, by converting them into electrical signals for processing and analysis [3]. Ideal sensors are imperative components of various measurement systems, possessing specific characteristics such as a wide operational range, minimal drift, ease of calibration, high sensitivity, and selectivity [4]. Sensor technology has gained importance in numerous fields, including environmental and food quality monitoring, medical diagnosis and healthcare, automotive and industrial manufacturing, space exploration, and national security [5]. The development of sensors has led to their use in diverse fields, and one particularly exciting area is biosensors. The term “biosensor” was coined by Cammann and its definition was established by IUPAC [6]. Biosensors detect biological processes, diagnose diseases, identify environmental contaminants, and aid in drug discovery. Biosensors function by detecting biomolecules such as proteins, DNA, RNA, enzymes, and hormones, providing valuable insights into health conditions, disease progression, and environmental security [7]. Promptly and accurately identifying biomarkers plays a crucial role in advancing early disease diagnosis and personalized medicine [8]. Biosensor development is continuously advancing with a focus on designing highly sensitive and selective devices for biomolecular detection. However, biosensors face many challenges. Their accuracy and specificity are hampered by complex biological samples and environmental interferences. Device performance in terms of temperature and humidity requires careful control [9]. Additionally, some biosensors have a limited lifespan owing to degradation and require regular calibration for sustained accuracy. Furthermore, the development and maintenance of biosensors are expensive because specialized materials and expertise are required [9]. Figure 1 shows a schematic diagram of the biosensor device used in this study. The biosensor operates via a series of steps involving the interaction of a target analyte with a bioreceptor, leading to the generation of a measurable signal. 

Ongoing research is focused on improving biosensor sensitivity, selectivity, and stability, paving the way for wider application in the future. From this perspective, the fabrication of biosensors has gained significant attention for advancement in device functionality and practical applications [11]. The quest for precise and efficient biosensor design and fabrication technique is paramount for unveiling the potential of “smart” biosensor systems [12]. Various biosensor technologies enable biomolecule detection, each with its own operating principle. Optical biosensors use light interactions with biomolecules to measure them, piezoelectric biosensors track mass changes upon biomolecule binding, surface plasmon resonance (SPR) biosensors detect refractive index changes caused by complex biomolecules, and electrochemical biosensors measure the electrical signals produced during biomolecule binding at the electrodes [13]. Electrochemical biosensors are promising because of their sensitivity, affordability, portability, biocompatibility, simplicity, and fast response [14]. A biochemical receptor captures the response of a biological process and transmits it to a transducer [14]. An ideal biosensor is independent of temperature and pH, recyclable, and specific [15]. Electrochemical biosensors consist of three electrodes: a reference electrode (RE), a counter electrode (CE), and a working electrode (WE) [16]. Electrical signal detection involves electrons that are generated or consumed, and it is categorized as potentiometric, cyclic voltammetric (CV), chronoamperometric (CA), differential pulse voltammetry (DPV), impedimetric, and linear sweep voltammetry (LSV) [17]. The sensing mechanism includes the interaction between the analyte and the pinhole surface of the WE to induce a redox reaction. To decrease the overpotential and fouling effect and improve the response, virgin electrodes have been modified with appropriate redox materials or stimulants [17]. To optimize the interaction, an electrode surface has been fabricated with various conducting nano and rare noble materials (Au, Pt, etc.) to facilitate faster electron movement in biosensors [18]. Different redox-active materials such as NPs, metal oxides, carbonaceous materials, polymers, macrocycles, and organic compounds have been employed on the electrode surface because of their large surface area, enhanced conductivity, optical properties, and biocompatibility [18]. However, biosensors’ long-term stability, reproducibility, and cost-effectiveness remain challenging issues [19]. In response, various hybrid materials such as graphene oxide (GO)–NPs hybrids [20], polymer–enzyme hybrids [21], carbon nanotube (CNT)–quantum dot hybrids [22], metal–organic framework hybrids [23], and nanostructured composite hybrids [24] have been extensively designed for the development of novel materials with exceptional properties. 

Organic-based macrocycles (phthalocyanine (Pc)/porphyrin) are considered reliable materials for signal amplification in electrochemical sensing owing to the simplicity of their synthesis and the ability to fine-tune their electronic properties through the substitution of axial/peripheral groups [25]. N4 macrocycle Pcs are interesting because of their unique electronic structure, substantial surface area, distinctive atomic structure, and properties that can be engineered by substituting various functional groups or metal ions [26]. Pc consists of a tetrapyrrole core similar to that of porphyrins in natural systems. This structural resemblance to the heme group in hemoglobin and chlorophyll in photosynthetic organisms is one of the reasons for their bioinspired appeal [26]. The central metal ion in Pc plays a vital role in tuning the catalytic properties. Transition metals, such as Fe, Co, Mn, and Ni, are commonly used as metal centers in Pcs [26]. Furthermore, the rigid and planar structure of Pcs provides stability and promotes efficient electron and ion transport during catalytic processes [27,28]. Additionally, their notable features include high selectivity and a specific size for miniaturized binding geometries, which reduce interference and ensure more accurate data [28]. Furthermore, it offers improved detection limits, enhanced sensitivity, and signal amplification for targeted molecules at minute concentrations, making it a versatile material for biosensing applications. However, the design and fabrication of hybrid materials ensures high stability in harsh environments, and their smooth integration with smart devices underscores the utilization of macrocyclic complexes in biosensor applications. 

In recent years, the design and fabrication of biosensors using hybrid materials containing Pcs with metal oxide or carbon composites has increased, owing to the large scope of functional operations [29]. Hybrid materials provide unique exploitation owing to the collaborative attributes of each component. Pcs contribute to tunable biocompatibility, leading to improved sensor response and easier processing during fabrication [30]. Carbon nanomaterials (CNs) substantially enhance the electrical conductivity and surface area of biosensors, allowing improved detection efficiency. The conductivity test results reveal that once 10% rGO is encapsulated into the polymeric network, it results in the highest amount of conductivity (1.716 × 10^−3^ S/cm) [31]. Furthermore, the incorporation of metal nanoparticles (MNPs) further enhances the sensing performance through their catalytic activity, signal amplification, and unique optical properties [31]. Hence, the deployment of Pc–metal oxide or polymer–metal–carbon (PMC) hybrids has led to the development of biosensors with superior sensitivity, enabling the detection of analytes at lower concentrations [32]. Additionally, PMC hybrids offer improved selectivity, meaning that they can distinguish between the target molecule and potential interferences. The use of these hybrid materials paves the way for the miniaturization of biosensors, making them smaller, more portable, and potentially more suitable for various biomedical applications. Despite these positive observations, ensuring long-term stability, biocompatibility, reproducibility, minimal potential toxicity of NPs, and developing cost-effective production methods, as well as fabricating PMC-based hybrid biosensors, remain paramount challenges [30,31,32,33,34,35,36]. In this review, we briefly introduce hybrid materials. The synthesis strategies, properties, and sensing mechanisms of the hybrid materials are also incorporated, and their potential for the detection of analytes, such as biomarkers, environmental pollutants, and food contaminants, is explained. Furthermore, we explore recent advancements in the development and application of PMC-based hybrid materials for biosensors, particularly the integration of sensor systems, and their translation into practical applications. Finally, the challenges and future directions of PMC-based hybrids for biosensors are discussed. This survey’s novelty remarkably adds to the field of electrochemical biosensors by providing an extensive combination of the mechanisms through which phthalocyanines improve sensor performance, detailing the integration procedures with conductive materials, and proposing solutions for current difficulties and challenges. By offering deeper insights into electron transfer processes, surface area improvisation, and specific interaction with MPcs and analytes, this work paves the way for future progress and advancements in MPc-based biosensor innovation technologies.

## 2. Properties of MPcs

MPcs are a group of N4 macrocyclic complexes with unique planar structural, electric, and optical characteristics, rendering them suitable for many electrochemical and electrocatalytic applications [13,16,17]. The MPcs possess exceptional planar structure, functionalization capacity, and excellent thermal and chemical stability, making them highly selective, sensitive, and stable for detecting various biomaterials in biosensor applications [13]. These materials have a planar macrocyclic ring made of metal in the middle and four isoindole subunits associated with four nitrogen atoms with delocalized π-conjugated electrons [31,37]. This property is a fundamental and crucial phenomenon for electrochemical biosensors; it ensures effective electron transfer between the biological sensor materials and the terminal surface of the MPcs, subsequently working on the sensor’s sensitivity and selectivity. The metal atom at the center of MPcs can undergo reversible redox reactions. This redox property allows MPcs to participate successfully in the electrochemical reaction process, improving the sensor’s capacity to recognize different biological and chemical analytes. For instance, the iron (II) and iron (III) MPc complexes can transfer electrons between various oxidation states, which enables the identification of substances through redox reactions. The adaptability of the high-charge transporter is attributed to its planar structure and extensive π-conjugation. This feature is fundamental for rapid and effective charge transmission, which is also essential for real-time sensing and detecting applications.

MPcs have strong absorption in the UV–vis and IR regions. This optical characteristic will be of considerable benefit to optical biosensors and the advancement of electrochemical signals through photoelectrochemical devices. Certain MPcs have photocatalytic properties, enabling them to generate reactive oxygen species (ROS) under light irradiation. This part can be utilized to degrade target analytes or improve biosensor sensitivity through light-prompted reactant processes. Few MPcs are fluorescent, which can be used in dual-mode recognition stages that join electrochemical and optical identification strategies. The fluorescence properties of MPcs can enhance specificity and sensitivity in biosensor applications. MPcs show high thermal and chemical stability, making them suitable for use in cruel ecological circumstances [16,17]. This power guarantees that MPc-based biosensors can keep up with their performance over extended periods of time and under various operational and functional conditions. Overall, the structure, electronic, and optical properties of MPcs make them significantly favorable for use in electrochemical biosensors. Their high conductivity, redox activity, and charge carrier mobility improve sensor electronic performance. Likewise, their strong photocatalytic activity, strong absorption characteristics, and fluorescence properties add to their work on optical and electrochemical signal transduction. The planar and conjugated construction, high thermal and electrochemical stability, ensure robust and strong sensing and detecting design, as well as extended practical and functional engaged lifetimes.

## 3. Mechanisms and Factors Influencing the Improved Performance of MPcs as Biosensors

MPcs have shown tremendous advantages and high performance as electrochemical biosensors. The mechanism behind the superior sensitivity, selectivity, and stability of these systems is elaborated in this section. The fundamental and essential mechanisms include the electron transfer process, increased surface area, and explicit interactions between MPcs and the analytes. The electron transfer properties are described by a delocalized π-conjugation electron, which works with quick electron transfer and directly impacts the sensor’s reaction and sensitivity [13,16,17]. Essentially, the central metal atom additionally assumes an emerging role in this process. Metal focuses can go through reversible redox responses; in this way, they upgrade the general electron transfer energy of the sensors, prompting high sensitivity and quicker reaction times. MPcs can act as redox mediators, connecting the electrons moving between the terminal and analyte. The redox-active locales in MPcs work with electron jumping. This enhancement improves the overall electrical conductivity and facilitates more efficient electrochemical signal transduction in the sensors. This enhanced impact is pivotal to achieving a lower detection limit (LOD) and further developing sensor performance.

The planar development of MPcs takes about a high surface-to-volume extent, which is basic for biosensor applications. A bigger surface area gives more powerful areas for biomolecule immobilization, like impetuses, antibodies, or DNA strands. This extended immobilization limit works on the association between the biosensor and the goal analyte, empowering further sensitivity advancement. When combined with nanomaterials such as carbon nanoparticles, carbon nanotubes (CNTs), reduced graphene oxide, and graphene, the metal phthalocyanine complex benefits from the high surface area and high conductive properties. MPcs and nanomaterials work together to make crossover composites that have synergistic effects. The nanomaterials add structure support and more active surface areas, while the MPcs improve the electron transfer processes. This coordination leads to significant improvements in biosensors’ electrochemical performance.

The MPcs metal or central metal atom can be customized to associate specifically with explicit analytes. This limitation is facilitated by the coordination chemistry of the metal atom, which can form specific interactions with specific particles. For instance, copper phthalocyanine (CuPc) has a high affinity for nitrogen-containing compounds, making it particularly significant for recognizing such analytes. In contrast, Cobalt phthalocyanines (CoPc) are known for their catalytic properties in oxygen reduction reactions, which can be leveraged for detecting oxygen-containing analytes. These correspondences work on the selectivity of the biosensor, ensuring that the signal delivered is unique considering the target analyte. MPcs can be functionalized to further develop their molecular recognition capacities. Functional groups can be attached to the peripheral destinations of the MPc particles, which collaborate expressly with the objective analytes. These cooperations can be hydrogen bonds, van der Waals powers, or π-π stacking coordinated efforts. The atomic recognition properties of functionalized MPcs add to the high selectivity and sensitivity of the biosensors. The consideration of MPcs in biosensor designs additionally adds to the sensors’ stability. MPcs are known for their high thermal and synthetic dependability, which ensures that the biosensors can work effectively under a wide range of circumstances. This stability is basic for practical applications where the sensors may be exposed to changing environmental conditions.

## 4. Pc Composite Materials for Biosensors

### 4.1. Polymeric Pc Materials

**Figure 2:**
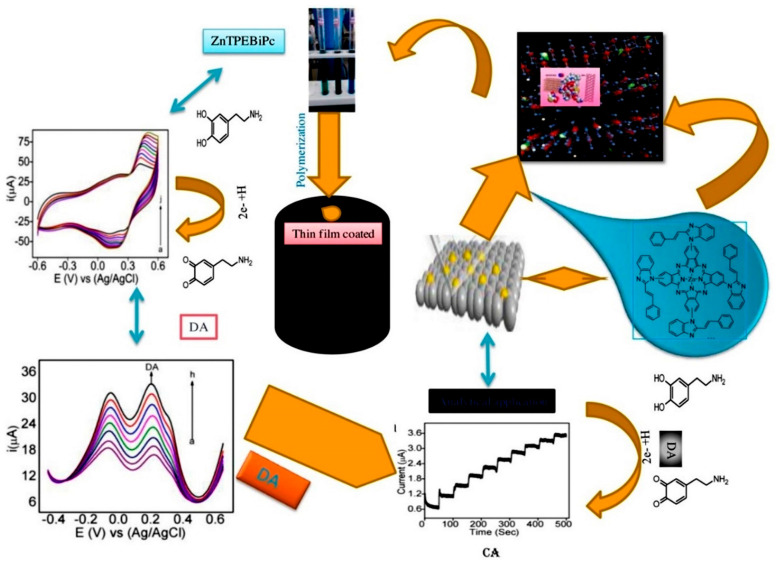
Schematic representation, modification of electrode and mechanism for detection of DA using an interface made with a thin film of polymeric zinc(II) Pc. Reproduced with permission [43]. Copyright 2020 from Elsevier Publications, Amsterdam, The Netherlands.

Polymeric Pcs are a group of aromatic macrocyclic polymers known for their unique electronic properties, rich redox behavior, strong absorption capability, tunable biocompatibility, and high thermal and electrochemical stabilities in corrosive media [38]. These outlined electrocatalytic properties contribute to the high efficacy of Pc/porphyrin and its analogs, which are similar to naturally occurring porphyrin macrocycles such as vitamin B-12, hemoglobin, cytochrome-c, and chlorophyll [39]. Several MPcs, such as ZnPc, CoPc, RhPc, NiPc, and TiPc, have been widely employed in dyes and pigments, photovoltaic devices, electronics, photodynamic therapy, sensors, and biosensors [40]. Pcs are used to enhance the sensitivity and selectivity of biosensors. For instance, Pcs act as mediators in redox reactions, facilitate electron transfer and improve biosensor efficiency [41]. CoPc is widely used as a redox mediator in enzymatic glucose biosensors. Additionally, the catalytic activity, stability, and electrochemical sensitivity of CoPc contribute to its overall performance, making it the most preferred candidate for biosensors. However, ZnPc faces instability in biosensors owing to its weak electrochemical activity and low electrical conductivity [42]. Therefore, graphene nanosheets and CNTs have been incorporated into MPcs to enhance electron transfer and improve their electrical conductivity [42]. M. Pari et al. (Figure 2) demonstrated the effective detection of DA using a composite of rGO and zinc tetra [4-{2-[(E)-2-phenylethenyl]-1H-benzimidazol-1-yl}] Pc (Zn(II)TPEBiPc). The rGO-Zn(II)TPEBiPc composite-modified electrode showed better performance compared to Zn(II)TPEBiPc film, with a detection limit of 6 nM and sensitivity of 2.8784 μA μM^−1^ cm^−2^ for DA detection in the range of 20 nM to 1.0 μM. Furthermore, it exhibited good stability and repeatability and was successfully applied for DA detection in pharmaceutical drugs [43]. Similarly, S. Malali et al. worked on graphene-based biosensors for selective DA detection using hybrid tetra-amino cobalt (II) Pc (TACoPc) and polyaniline (PANI) nanofibers (TACoPc/PANI hybrid). The hybrid synthesized via a one-step process exhibited superior DA detection performance compared to conventional methods. The TACoPc/PANI hybrid-modified electrode displayed a high sensitivity of 1.212 μA μM^−1^ cm⁻^2^ and a low detection limit of 0.064 μM for DA within a 20–200 μM concentration range in a phosphate buffer solution with pH 7. The synergistic effect of PANI and TACoPc eliminated the interference from ascorbic acid (AA), which is a major challenge for DA detection [44]. Sajjan et al. [45] designed the peripheral amine of Pc to form a polymeric film on the electrode surface in DMSO. The polymeric film-modified electrode exhibited excellent voltammetric and amperometric detection of DA, with linear responses of 100–4000 nmol L^−1^ and 100–1000 nM, respectively. Additionally, the amperometric analysis displayed a high correlation coefficient (R2 = 0.999), low LOD (20 nmol L^−1^), and good sensitivity (0.024 μA nmol^−1^ cm^−2^).

**Figure 3:**
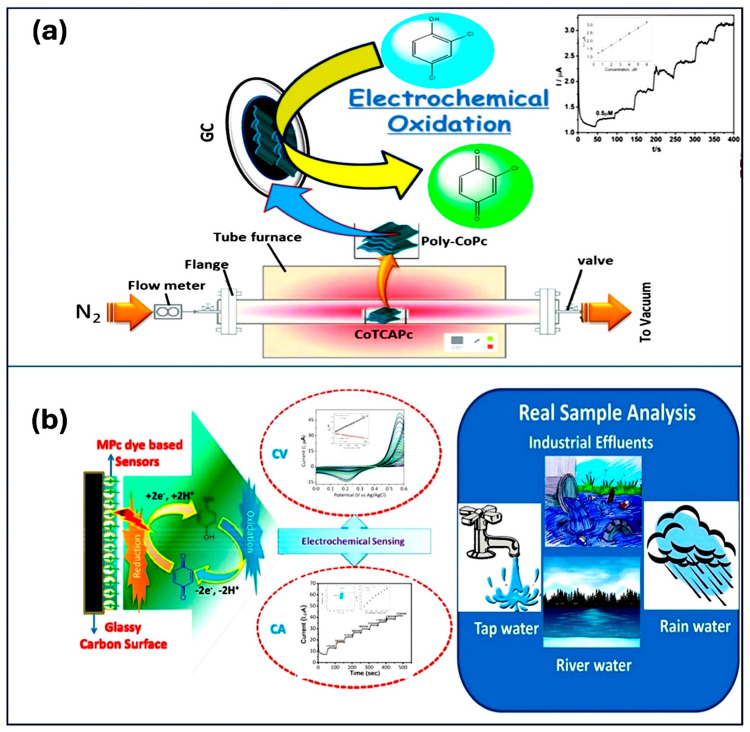
(**a**) Design and fabrication of cobalt phthalocyanine sheet polymer (poly-CoPc) for the detection of 2,4-dichlorophenol (DCP). Reproduced with permission [46]. Copyright 2020 from Elsevier Publications. (**b**) Electrocatalysis using amide coupled Pcs for detection of hydroquinone. Reproduced with permission [47]. Copyright 2021 from Elsevier Publications.

Imadadulla et al. [46] synthesized a cobalt Pc sheet polymer (poly-CoPc) through the thermal condensation of cobalt tetracarboxylic acid Pc (CoTCAPc) and investigated for 2,4-dichlorophenol (DCP) analyte (Figure 3a). The polymer exhibited high thermal stability (up to 420 °C) and redox-active behavior and was successfully immobilized on GCE (GC/poly-CoPc). Voltammetric analysis revealed a linear response for DCP in the concentration range of 1–36 µM with an LOD of 0.35 µM and a sensitivity of 0.08 µA µM^−1^. Amperometric measurements showed a linear response for DCP between 0.5 and 10 µM, with an even lower LOD of 0.15 µM and a sensitivity of 0.0384 µA µM^−1^. The sensor displayed excellent stability, repeatability, reproducibility, and high selectivity toward DCP, even in the presence of various other alcohols. These results suggest that poly-CoPc holds promise as a sensitive and selective electrochemical sensor for DCP. Nemakal et al. investigated amide-bridged cobalt Pc (CoTAMFCAPc) complexes for hydroquinone sensing and confirmed that the furan-containing complex had superior performance [47]. Same group investigated the nanomolar detection of lead using electrochemical methods based on a novel CoTBrIMPPc complex. The GCE/CoTBrIMPPc modified electrode showed 500–3000 nmolL^−1^ linear range and 180 nmolL^−1^ LOD with sensitivity of 0.0035 µA nM^−1^ [48]. Similarly, Shantaraj et al. [49] designed a novel cobalt (II) Pc polymeric material (poly-CoTPzPyPc) for the detection of l-arginine. The designed material exhibited a linear response to l-arginine concentration in the range of 10–100 μM with an LOD of 2.5 μM. The diffusion coefficient for l-arginine was calculated as 1.67 × 10^−6^ cm^2^/s. The rotating disc electrode performance confirmed the 2e^−^-transfer process during l-arginine oxidation. CA studies showed the catalytic response for l-arginine in the range of 2–60 μM with an LOD of 0.6 μM. The poly-CoTPzPyPc film demonstrated excellent selectivity toward l-arginine in the presence of biomolecules. Furthermore, the sensor displayed good stability and satisfactory performance in the analysis of real samples. These findings suggest that poly-CoTPzPyPc has the potential to detect and monitor l-arginine in biological samples. Sajjan et al. [50] further explored the remarkable sensing capabilities of a GCE/CoTTIMPPc electrode toward 4-nitrophenol. Keshavandaprabhu et al. [51] synthesized a dark blue cobalt (II) Pc complex (CoTBrImPc). The complex exhibited promising electrocatalytic activity toward L-cysteine detection. Immobilized on GCE, it demonstrated excellent performance with a low detection limit of 3 nM and a high sensitivity of 2.99 μA nM⁻^1^ cm⁻^2^. Notably, the linear response ranged from 10 to 100 nM, indicating the potential application of CoTBrImPc in L-cysteine sensing. These advancements highlight the versatility and potential of polymeric Pc-based materials in developing next-generation sensors for various analytes (Figure 3b). Table 1 summarizes the diverse applications and performance characteristics of polymeric Pc materials in the detection of various analytes. Each entry in the table highlights the specific target analyte, the detection method employed, the sensitivity, detection limit, and advantages of each material. This underscores the versatility and effectiveness of polymeric Pcs in analytical sensing applications [43,44,45,46,47,48,49,50,51].

### 4.2. Pc-Based Hybrid Composites

In recent years, the integration of an organic hybrid composite of Pc with metal oxides or CNs in biosensors has shown promise owing to the unique combination of properties and synergistic framework. Pc provides tunable biocompatibility and enhances sensor sensitivity and processability, whereas CNs (CNTs, graphene, and GO) significantly enhance the electrical conductivity of the composite materials, thereby improving the overall sensor performance and a large surface area [52]. MNPs exhibit catalytic activity, signal amplification, and unique optical properties that further enhance the sensing competence of hybrid materials [53]. The integration of PMC-based hybrids reinforces the performance of biosensors with improved sensitivity, detection limit, selectivity, multifunctionality, and miniaturization [54]. The improved biosensing performance of hybrid composites is explained below.

(a) Combining MPc with carbon materials in a hybrid composite overcomes the MPc aggregation issue and enhances biosensor sensitivity [55].
(b) An 18 п-electron system in MPc combined with carbon materials results in the optimization of hybrid composites with high porosity, surface area, and conductivity. This contributes to improved specificity in detecting biomolecules and enhances selective detection [56].
(c) The integration of carbon materials into a hybrid composite not only enhances electrical conductivity and electron mobility but also facilitates efficient electron transfer, ensuring a rapid biosensor response [57,58].
(d) Carbon materials in hybrid composites solve the stacking arrangement issue in MPc and improve dispersion, diffusion, and adsorption to overcome issues related to long-term stability [59].
(e) The immobilization of the hybrid composite on the electrode surface in a hierarchical structure leads to strong affinity and stability, thus contributing to an efficient biosensing process [9,60].

**Figure 4:**
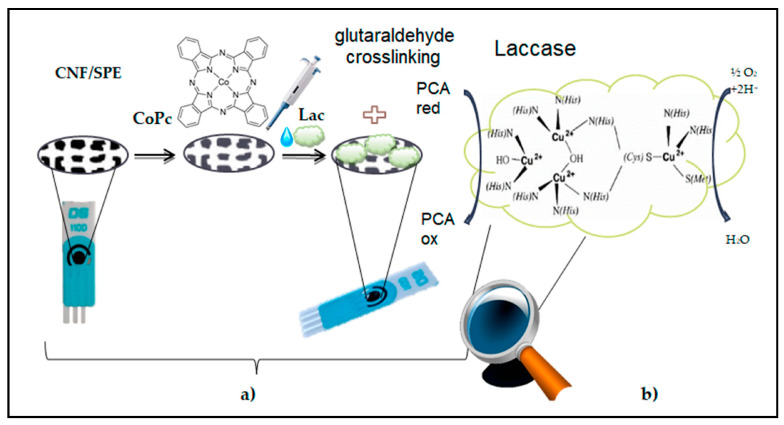
(**a**) Fabrication of a laccase-based biosensor on an SPE, based on CoPc-modified CNFs. (**b**) The enzymatic oxidation mechanism of PCA in the presence of laccase [61].

Figure 4 illustrates the step-by-step preparation process of a laccase-based biosensor on a screen-printed electrode (SPE) modified with cobalt Pc-modified carbon nanofibers (CoPc-CNFs). The fabrication procedure involves several key steps. Carbon nanofibers are initially treated to enhance their surface properties and facilitate the binding of the cobalt Pc molecules. Cobalt Pc, known for its catalytic properties and stability, is then immobilized onto the surface of the carbon nanofibers through a suitable deposition method, such as drop-casting or electrodeposition. An SPE is utilized as the substrate for the biosensor owing to its ease of fabrication, portability, and compatibility with different detection techniques. The SPE is fabricated by printing a layer of conductive ink onto a substrate and pasting CoPc-CNFs onto the WE surface area. The laccase enzyme has the ability to catalyze the oxidation of phenolic compounds immobilized on the CoPc-CNFs-modified electrode surface. The immobilization process involves incubating the electrode in a laccase-containing solution under controlled conditions, allowing the enzyme to adsorb or bind to the electrode surface. The enzymatic oxidation mechanism of protocatechuic acid (PCA) in the presence of laccases is shown in Figure 4b. Laccase catalyzes PCA oxidation by transferring electrons from the substrate to molecular O_2_, resulting in the formation of reactive intermediates that lead to the formation of quinone products. In the oxidation process, electrons are transferred from PCA to the laccase enzyme, which undergoes a redox cycle involving the active sites of copper ions (Cu^2+^ and Cu^3+^), facilitating the oxidation reaction. This enzymatic oxidation of PCA leads to the formation of a quinone product via the release of protons and electrons. Figure 4 provides valuable insights into the fabrication and enzymatic oxidation mechanism of a laccase-based biosensor for the detection of phenolic compounds such as PCA. The integration of CoPc-modified CNFs for laccase immobilization enhances catalytic activity and sensitivity in biosensor applications.

Additionally, the incorporation of carbon materials like graphene or nanotubes disrupts π-π stacking in Pcs, which hinders the accessibility of active sites for biomolecule interaction. The improved dispersion maximizes the surface area for biomolecule adsorption during sensor fabrication. Furthermore, the 18 π-electron system of MPcs interacts with the carbon network to optimize the electronic structure of the composite. This enhanced π-conjugation facilitates efficient electron transfer and improves interfacial charge transfer between the immobilized biomolecule and the composite, leading to a superior detection level of Hydrazine (Hz). Additionally, the excellent conductivity of carbon materials significantly increases the overall conductivity of the composite, resulting in a fast response and increased sensitivity during fabrication. Moreover, the optimized structure during fabrication maximizes the surface area for biomolecule interaction, which promotes strong binding with target biomolecules and facilitates efficient mass transport, thereby boosting the sensitivity and response time of the biosensor [62]. 

**Figure 5:**
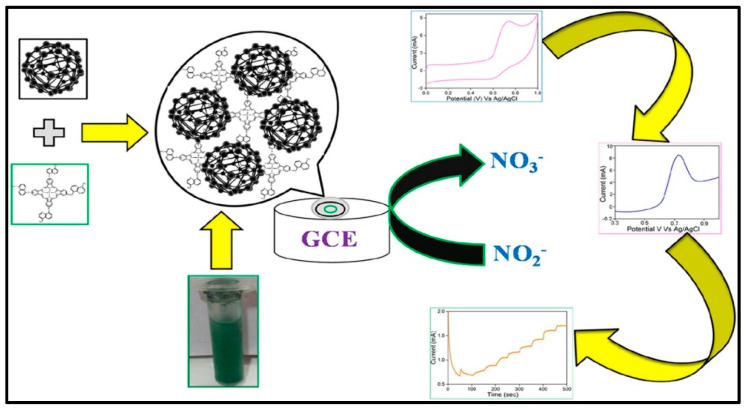
Investigation of a GCE modified with cobalt (II) tetra methyl-quinoline oxy-bridged Pc and carbon NPs for the detection of nitrite. Reproduced with permission [63]. Copyright 2020 from Elsevier Publications.

**Figure 6:**
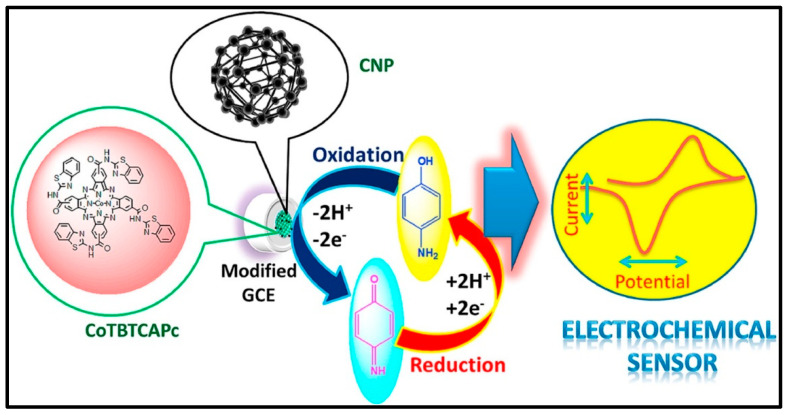
Hybrid Pc-based amperometric sensor for nanomolar detection of 4-AP. Reproduced with permission [65]. Copyright 2019 from Elsevier Publications.

Hybrid composites of rGO have gained attention for biosensing applications because of their unique properties. rGO provides high conductivity and a large surface area, facilitating efficient electron transfer and increasing biomolecule interaction. This translates into potentially faster and more sensitive biosensors, aiding in the development of high-performance biosensors with modified electrode surfaces that exhibit minimal interfacial resistance, exceptional stability, and efficient electron transfer between the electrolyte and electrode. Recently, Jilani et al. [63] explored the potential of carbonaceous and metal phthalocyanine (MPc) hybrid composites for nitrite-sensing applications. As shown in Figure 5, they synthesized a novel composite material, cobalt (II) tetramethylquinoline oxy-bridged Pc (CoTM-QOPc), and used it for nitrite sensing. The sensor exhibited a linear detection range of 0.3 to 120 μmol/L, using CV, and 0.2 to 170 μmol/L, using CA. The detection limit achieved was 0.1 μmol/L for CV and 0.06 μmol/L for CA, with good sensitivity of 0.765 μA μM⁻^1^ cm⁻^2^ (CV) and 1.204 μA μM⁻^1^ cm⁻^2^ (CA). Recognizing the potential for further improvements, they strategically incorporated CNP into the CoTM-QOPc matrix. This carbonaceous and MPc hybrid composite (CoTM-QOPc/CNP) offered an enhanced electrocatalytic response for nitrite oxidation compared to the electrode modified with Pc alone. The linear detection range for nitrite sensing using the composite electrode was even broader, spanning from 0.2 to 200 μmol/L (CV), 0.2 to 225 μmol/L (DPV), and 0.1 to 350 μmol/L (CA). The detection limit also improved remarkably, reaching 0.06 μmol/L for both CV and DPV, and 0.033 μmol/L for CA. The sensitivity of the CoTM-QOPc/CNP composite electrode was also superior, with values of 2.298 μA μM⁻^1^ cm⁻^2^ (CV), 1.031 μA μM⁻^1^ cm⁻^2^ (DPV), and 1.237 μA μM⁻^1^ cm⁻^2^ (CA). This highlights the remarkable utilization of carbonaceous materials in Pc-based sensors. The CoTM-QOPc/CNP composite electrode demonstrated not only enhanced sensitivity and detection limits for nitrite but also impressive selectivity, even in the presence of interfering ions such as AA, carbonate, urea, phosphate, and glucose. This study paves the way for the development of highly selective and sensitive biosensors for real-world applications. Similarly, Shambulinga et al. [64] designed an oxy-bridged cobalt Pc polymer (poly(TazoCoPc)) to enhance the conjugation effect for nitrite detection. Furthermore, poly(TazoCoPc) doped with CNP was utilized for electrochemical voltammetric and amperometric nitrite sensors. The poly(TazoCoPc)/CNP composite demonstrated superior electrocatalytic activity for nitrite oxidation compared to pure poly(TazoCoPc). The amperometric sensor showed excellent performance in the detection of nitrite concentrations ranging from 20 nM to 1 μM with a detection limit of 6 nM and a sensitivity of 0.137 mA/μM. The modified electrode exhibited high selectivity with no interference from ions such as Mg^2+^, SO_4_^2−^, K^+^, CO_3_^2−^, and NO^3−^. Similarly, Manjunath et al. [65] designed a cobalt (II) tetra-β-[N(2-(1,3-benzothiazole)) carboxamide] Pc (CoTBTCAPc) for the detection of 4-aminophenol (AP) (Figure 6). The GCE/CoTBTCAPc electrode displayed poor charge transfer, whereas the composite electrode with CNP (GCE/CNP-CoTBTCAPc) showed improved charge transfer. Both electrodes exhibited reduced overpotential and increased oxidation peak current. For 4-AP detection in phosphate buffer, they exhibited linear responses with detection limits of 13 nM (GCE/CoTBTCAPc) and 11 nM (GCE/CNP-CoTBTCAPc). DPV showed sensitivities of 0.0328 and 0.4179 μA nM^−1^ cm^−2^, while amperometry showed sensitivities of 0.4008 and 0.8887 μA nM^−1^ cm^−2^, with LODs of 40 and 30 nM. The GCE/CNP-CoTBTCAPc electrode was selective for 4-AP in the presence of interferents, making it suitable for the analysis of real samples, such as 4-AP in paracetamol tablets. 

**Figure 7:**
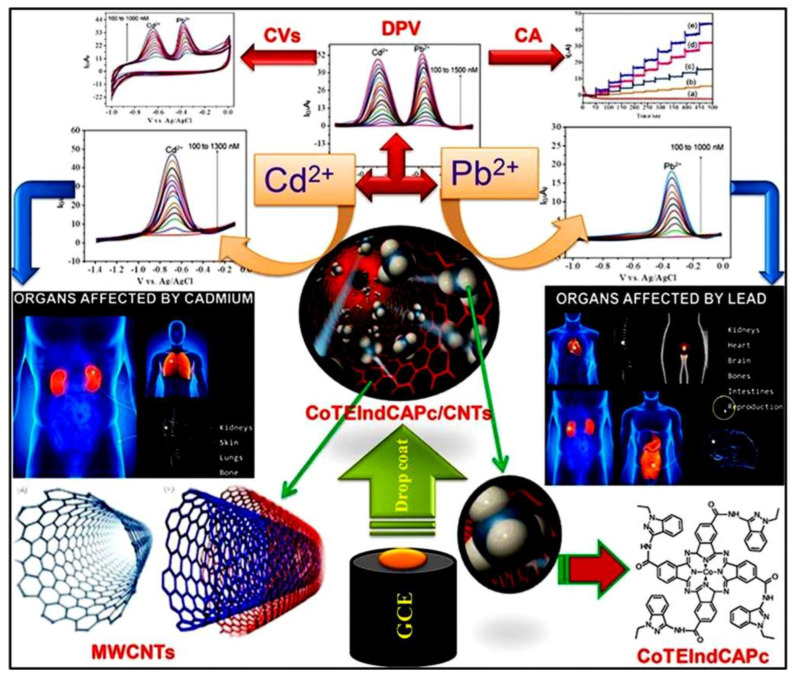
Graphical representation and mechanism of CoPc functionalized with MWCNTs/GCE for electrochemical detection of heavy metals (Cd^2+^ and Pb^2+^). Reproduced with permission [67]. Copyright 2021 from Elsevier Publications.

**Figure 8:**
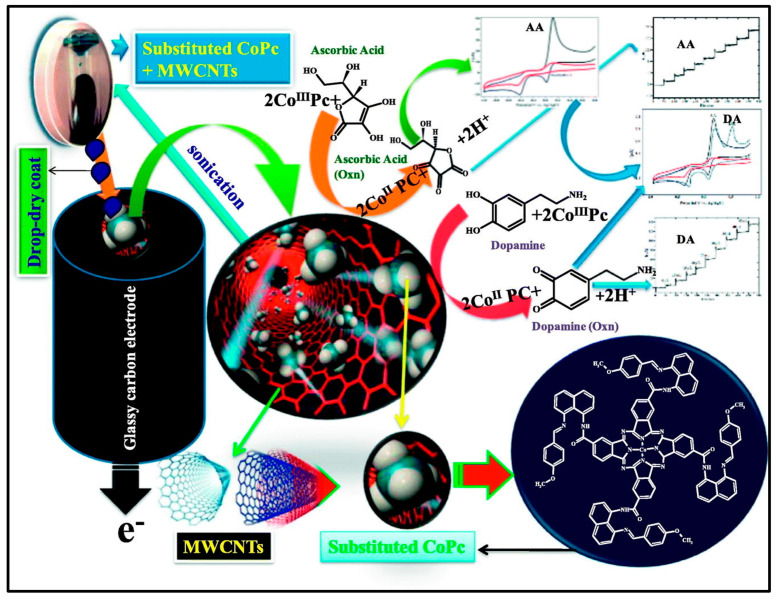
Electrochemical detection of AA and DA utilizing a GCE modified with a hybrid cobalt (II) Pc complex. The cobalt (II) Pc acts as a catalyst, enhancing the electron transfer between the target molecules (AA and DA) and the electrode surface, leading to a more sensitive and selective detection process. Reproduced with permission [68]. Copyright 2019 from Elsevier Publications.

CNs, particularly MWCNTs, have immense potential as next-generation biosensors. In Pc-based composites, MWCNTs improve conductivity and enhance electron transfer with faster response times and potentially higher sensitivity. Additionally, the MWCNTs increase the effective surface area by providing more space for biomolecule interactions and potentially more binding sites. These CNs contribute to the overall stability of the composite by offering mechanical support, leading to a more robust and long-lasting biosensing activity. Hence, the synergistic effects of incorporating MWCNTs into Pc composites provide great possibilities for the development of superior biosensors with enhanced sensitivity, stability, and performance [66,67]. The electrocatalytic performance of a CoTEIndCAPc/MWCNTs/GCE electrode for Cd^2^⁺ and Pb^2^⁺ detection is illustrated in Figure 7. This figure illustrates the schematic diagram, sensor mechanism, structure of MWCNT, CoTEIndCAPc materials and indicate the organs affected by cadmium and lead. The electrode was designed and utilized for CV, DPV, and CA measurements of electrochemical activity. The electrode showed excellent sensitivity with low detection limits of 10 nmol L⁻^1^ for Cd^2^⁺ and 9 nmol L⁻^1^ for Pb^2^⁺, and high reproducibility, highlighting its potential for biological applications [67]. Recently, Mounesh et al. [68] presented an intriguing approach for biosensing applications using carbonaceous and MPc hybrid composites. They synthesized a novel tetra-8-[(E)-(4-methoxybenzylidene) amino] naphthalene-1-amine cobalt (II) Pc (CoTMBANAPc) through an amide-bridge linkage, using cobalt (II) tetracarboxylic acid Pc (CoTCAPc) as the initial material. This synthesized macromolecule displayed excellent solubility in aprotic organic solvents, providing valuable insights into the composition and structure of the material. The GCE/MWCNT-CoTMBANAPc electrode exhibited remarkable promise for the simultaneous detection of AA and DA using CV, DPV, and CA techniques. The detection performance was within the linear response of the concentration range of 7.5 to 70 nM for both AA and DA. The composite exhibited a lower detection limit of 6.6 μM for AA and 0.33 nM for DA. Furthermore, the GCE/MWCNT-CoTMBANAPc electrode displayed excellent stability, sensitivity, and reproducibility within the micromolar range. However, exploring the selectivity of these sensors in complex biological matrices where additional interfering species are present is necessary. Keshavanand Prabhu et al. [69] developed FeTBImPc, modified it with CNP, and immobilized it on a GCE for the detection of DA. The composite electrodes (GCE/CNP-FeTCAPc and GCE/CNP-polyFeTBImPc) showed excellent electrocatalytic activity toward DA oxidation, with lower detection limits of 14 nM. The GCE/CNP-polyFeTBImPc sensor exhibited superior performance with a high sensitivity of 67.2039 mA nM⁻^1^ cm⁻^2^. However, carbonaceous and metal-Pc hybrid composites face many challenges. The aggregation of Pc molecules limits the available surface area for biomolecule interaction and hinders sensor performance. The long-term stability of the composites under real-world conditions requires further investigation. Figure 8 provides a comprehensive overview of the applications of hybrid Pc composites as biosensors. The cobalt (II) Pc-modified GCE exhibited enhanced performance for the detection of AA and DA. Table 2 highlights the versatility of various hybrid Pc composites in terms of their sensitivity and selectivity [62,63,64,65,66,67,68,69,70,71].

## 5. Fabrication of Hybrid Pc in Three-Electrode System

The fabrication of hybrid Pc materials for electrochemical applications using a three-electrode system involves several advanced techniques aimed at significantly enhancing the conductivity, stability, and catalytic efficiency of the WE [72,73,74,75]. In a typical three-electrode setup, the WE is where the electrochemical reaction of interest occurs, the CE completes the electrical circuit, and the RE maintains a stable potential, allowing for precise measurement of the potential and current of the WE. Thin film deposition techniques, such as chemical vapor deposition (CVD) and sputtering, are essential for achieving precise control over film thickness, consistency, and uniformity. In CVD, volatile precursors decay and react on the substrate, forming a thin, uniform film of Pc, which is crucial for the formation of deformity-free layers and effective electron transfer. Faltering involves the physical injection of material from an objective through high-energy ion bombardment. The material is then deposited onto the WE surface, providing a controlled and uniform coating that is fundamental for steady electrochemical performance. Electrochemical deposition further customizes the WE by integrating different metal ions into the electrode surface, thus fitting the catalytic properties of Pcs. This technique involves immersing the WE in an electrolyte containing metal ions and applying a voltage, which reduces the metal ions and deposits them on the electrode surface. This technique is particularly beneficial for framing nanostructured surfaces that significantly enhance catalytic activity and stability. Furthermore, self-gathering procedures, including the development of self-assembled monolayers (SAMs), are utilized to create coordinated molecular structures on the WE. SAMs involve immersing the WE in a solution containing particles with a functional group that binds strongly to the electrode material. These molecules spontaneously form a monolayer, providing a coordinated design that improves charge energy kinetics and sensitivity by ensuring that the Pc molecules are optimally oriented for electron transfer.

Hybridization with nanomaterials, such as graphene, CNTs, rGO, and MNPs, is achieved through techniques such as solution-phase mixing, in situ development, or layer-by-layer assembly. This integration significantly expands the surface area and conductivity of the WE, leading to improved electron transfer rates and electrochemical performance. The collaboration between Pcs and nanomaterials results in composite materials with superior electron transfer rates and enhanced strengths under electrochemical conditions. Advanced lithographic methods, including photolithography and electron beam lithography, are used to design the WE with high accuracy, facilitating the formation of complex and miniaturized electrode designs. Photolithography uses light-sensitive photoresists to achieve micron-level accuracy, whereas electron beam lithography offers considerably greater resolution for nanoscale fabrication. These techniques ensure the optimization of the cathode surface for high electrochemical activity, making hybrid Pc materials ideal for various applications. Using these fabrication techniques, researchers can optimize the WE interface in a three-electrode system framework, thereby significantly enhancing the performance and reliability of electrochemical biosensors.

## 6. Conclusions, Challenges, and Future Perspectives

This manuscript highlights the exciting potential of macrocycle–macromolecule hybrids for biomolecule sensor applications. These hybrids offer excellent durability, precision, and sensitivity, overcoming difficulties and challenges such as redox behavior, cost, and synthetic complexity. However, further advancements are expected to achieve unrivalled sensitivity and ultra-low detection limits, particularly in intricate biological matrices. The careful selection of reactants with flexible functional groups during macromolecule synthesis is critical for sensor power against interference and environmental variations. Real-time biomolecule detection in different medical conditions is fundamental for the development of portable and remote biosensors. The resolution of biocompatibility and stability issues, combined with the ability to functionalize these hybrid sensors for multi-analyte identification, holds enormous potential for customized medical healthcare, surpassing traditional diagnostic strategies. Optimizing the analyte–electrode interface and sensor variability is key to addressing biocompatibility, stability, and reproducibility concerns. Investigating novel materials, nanostructures, and coatings offers promising ways to enhance accuracy and efficiency. The incorporation of progressive nanostructures appears to be particularly encouraging for advancing biosensor innovation. These nanostructures offer unique benefits, such as increased surface area and improved signal transduction, ultimately resulting in superior sensitivity, extremely low detection limits, and improved noise control in biosensing applications. Standardization initiatives and regulatory cooperation are crucial for effective clinical collaboration. A multidisciplinary approach that coordinates expertise in materials science, biotechnology, and engineering is fundamental for fully understanding the capability and potential of biosensing platforms and improving global health conditions. 

Despite the comprehensive nature of this review, there are several limitations and a few impediments to be recognized. First and foremost, the focus has predominantly been on specific types of biosensors, especially those utilizing carbon nanomaterials and metal nanoparticles related to Pcs. This concentration, while important, may neglect other promising hybrid composite systems that could additionally upgrade biosensor performance. Also, the audit primarily examines current advancements without delving deeply into a few emerging areas where Pcs could have significant effects. For example, the coordination of MPc-based materials with novel nanomaterials or advanced manufacturing strategies has not been broadly covered. Future research aims to address these gaps by investigating diverse sensor types, extending the scope of hybrid materials, and researching the utilization of MPcs in novel and emerging biosensing advances. Further investigations could provide a more holistic understanding of the potential of MPc-based biosensors and drive development in the field.

## Figures and Tables

**Table 1:** Parameters of polymeric Pc materials for the detection of various analytes.

Materials	Method	Analyte	LOD	Linear Range	Ref.
Zn(II)TBPc	Amperometry	DA	6 nM	20 nM–1.0 μM	[43]
TACoPc/PANI	CV	DA	0.064 μM	20–200 μM	[44]
Poly(CoTNBAPc)	Amperometry	DA	20 nmol/L	100–4000 nM/L	[45]
	CV		20 nmol/L	10–1000 nM	
CoTCAPc	CV	2,4-dichlorophenol	0.35 μM	1–36 μM	[46]
CoTAMFCAPc/GCE	CV	Hydroquinone	0.066	0.2–2.2 μM	[47]
	Amperometry		0.056	0.17–1.530 μM	
Poly(CoTBrIMPPc)	CV	Pb	37 nmol/L	10–1000 nM	[48]
	Amperometry		180 nmol/L	500–3000 nM/L	
Poly(CoTPzPyPc)	CV	L-arginine	2.5 μM	10–100 μM	[49]
	Amperometry		0.6 μM	2–60 μM	
CoTTIMPPc	CV	4-nitrophenol	38 nM	100–1000 nM	[50]
	Amperometry		30 nM	100–900 nM	
Poly(CoTBrImPc)	CV	L- Cysteine	3 nM	10–100 nM	[51]
	Amperometry		4 nM	10–80 nM	

**Table 2:** Hybrid Pc composites for detection of various analytes.

Material	Method	Analyte	LOD	Linear Range	Ref.
rGO/Poly(CoOBImPc)	CV	Hz	0.033 μM	0.1–0.9 μM	[62]
CoTM-QOPc/CNP	CV	Nitrite	0.033 μM	0.1–350 μM	[63]
Poly(TazoCoPc)/CNP	CV	Nitrite	0.006 μM	0.02–1 μM	[64]
CoTBTCAPc/CNP	CV	4-AP	0.030 μM	0.1–0.9 μM	[65]
CoTELndCAPc/MWCNT	CV	Cd(II)	5 nM	100–1000 nM	[66]
		Pb(II)	3 nM	100–1000 nM	
PdTAPc/MWCNT	CV	AA	1.0 μM	3–24 μM	[67]
		DA	0.6 μM	2–16 μM	
CoTMBANAPc/MWCNT	CV	AA	6.6 μM	7.5–70 μM	[68]
Poly(FeTBImPc)/CNP	CV	DA	20 nM	100–1000 nM	[69]
RGO-pTACoPc	CV	L-cysteine	0.018 μM	0.05–2.0 μM	[70]
MWCNT-PNF	CV	Adenine (AD)	9.2 μM	0.01–3.9 mM	[71]
	DPV	AD	7.9 μM	0.01–7.9 mM	
	CV	Thymine (THY)	19.3 μM	0.02–7.7 mM	
	DPV	THY	16.8 μM	0.02–15.7 mM	
	CV	Guanine (GU)	98.56 μM	0.1–8.5 mM	
	DPV	GU	96.84 μM	0.1–3.5 mM	

## References

[1] Naresh V., Lee N. (2021). A Review on Biosensors and Recent Development of Nanostructured Materials-Enabled Biosensors. Sensors.

[2] Kulkarni M.B., Ayachit N.H., Aminabhavi T.M. (2022). Biosensors and Microfluidic Biosensors: From Fabrication to Application. Biosensors.

[3] Abid H., Mohd J., Ravi P.S., Suman R., Rab S. (2021). Biosensors applications in medical field: A brief review. Sens. Int..

[4] Mujawar M.A., Gohel H., Bhardwaj S.K., Srinivasan S., Hickman N., Kaushik A. (2020). Nano-enabled biosensing systems for intelligent healthcare: Towards COVID-19 management. Mater. Today Chem..

[5] Katey B., Ioana V., Anita N.P., Alexandrina U. (2023). A Review of Biosensors and Their Applications. ASME Open J. Eng..

[6] Thevenot D.R., Toth K., Durst R.A., Wilson G.S. (1999). Electrochemical biosensors: Recommended definitions and classification. Pure Appl. Chem..

[7] Singh A., Sharma A., Ahmed A., Sundramoorthy A.K., Furukawa H., Arya S., Khosla A. (2021). Recent Advances in Electrochemical Biosensors: Applications, Challenges, and Future Scope. Biosensors.

[8] Shivalingayya, Preeti R.K., Sangappa K.G., Shashidhar, Ashajyoti C., Arunkumar L. (2022). Multifunctional Nanoparticles for Biomedical Applications. J. Chem. Biol. Phys. Sci..

[9] Costa P., Nunes-Pereira J., Oliveira J., Silva J., Moreira J.A., Carabineiro S.A.C., Buijnsters J.G., Lanceros-Mendez S. (2017). High-performance graphene-based carbon nanofiller/polymer composites for piezoresistive sensor applications. Compos. Sci. Technol..

[10] Campaña A., Florez S., Noguera M., Fuentes O., Ruiz P.P., Cruz J., Osma J. (2019). Enzyme-based electrochemical biosensors for microfluidic platforms to detect pharmaceutical residues in wastewater. Biosensors.

[11] Lee J., Kim J., Kim S., Min D.H. (2016). Biosensors based on graphene oxide and its biomedical application. Adv. Drug Deliv. Rev..

[12] Basova T.V., Ray A.K. (2020). Review—Hybrid materials based on phthalocyanines and metal nanoparticles for chemi-resistive and electrochemical sensors: A Mini-Review. ECS J. Solid State Sci. Technol..

[13] Kuntoji G., Kousar N., Gaddimath S., Koodlur Sannegowda L. (2024). Macromolecule–Nanoparticle-Based Hybrid Materials for Biosensor Applications. Biosensors.

[14] Soto D., Orozco J. (2022). Hybrid Nanobioengineered Nanomaterial-Based Electrochemical Biosensors. Molecules.

[15] Huai-Song W. (2017). Metal–organic frameworks for biosensing and bioimaging applications. Coord. Chem. Rev..

[16] Keshavananda Prabhu C.P., Aralekallu S., Palanna M., Sajjan V., Renuka B., Sannegowda L.K. (2022). Novel polymeric zinc phthalocyanine for electro-oxidation and detection of ammonia. J. Appl. Electrochem..

[17] Manjunatha N., Lokesh K.S. (2021). Hybrid composites based on phthalocyanine and carbonaceous materials for sensing applications: A review. Int. J. Biosens. Bioelectron..

[18] Jipei Y., Nikolai G., Alexander E. (2012). Application of Polymer Quantum Dot-Enzyme Hybrids in the Biosensor Development and Test Paper Fabrication. Anal. Chem..

[19] Skladal P., Mascini M. (1992). Sensitive detection of pesticides using amperometric sensors based on cobalt phthalocyanine-modified composite electrodes and immobilized choline esterases. Biosens. Bioelectron..

[20] Lv N., Li Q., Zhu H., Mu S., Luo X., Ren X., Liu X., Li S., Cheng C., Ma T. (2023). Electrocatalytic Porphyrin/Phthalocyanine-Based Organic Frameworks: Building Blocks, Coordination Microenvironments, Structure-Performance Relationships. Adv. Sci..

[21] Aggas J.R., Guiseppi-Elie A. (2020). Responsive Polymers in the Fabrication of Enzyme-Based Biosensors. Biomaterials Science.

[22] Pourmadadi M., Rahmani E., Rajabzadeh-Khosroshahi M., Samadi A., Behzadmehr R., Rahdar A., Ferreira L.F.R. (2023). Properties and application of carbon quantum dots (CQDs) in biosensors for disease detection: A comprehensive review. J. Drug Deliv. Sci. Technol..

[23] Zhang Z., Lou Y., Guo C., Jia Q., Song Y., Tian J., Zhang S., Wang M., He L., Du M. (2021). Metal–organic frameworks (MOFs) based chemosensors/biosensors for analysis of food contaminants. Trends Food Sci. Technol..

[24] Basova T. (2024). Phthalocyanine and Porphyrin Derivatives and Their Hybrid Materials in Optical Sensors Based on the Phenomenon of Surface Plasmon Resonance. Chemosensors.

[25] Sorokin A.B. (2013). Phthalocyanine metal complexes in catalysis. Chem. Rev..

[26] Zagal J.H., Bedioui F., Dodelet J.P. (2007). N4-Macrocyclic Complexes.

[27] Hong Q., Chen S. (2020). Facile one-step fabrication of phthalocyanine–graphene–bacterial–cellulose nanocomposite with superior catalytic performance. Nanomaterials.

[28] Song Y., Meng Y., Chen K., Huang G., Li S., Hu L. (2024). Novel electrochemical sensing strategy for ultrasensitive detection of tetracycline based on porphyrin/metal phthalocyanine-covalent organic framework. Bioelectrochemistry.

[29] Gaddimath S., Payamalle S., Channabasavana Hundi Puttaningaiah K.P., Hur J. (2024). Recent Advances in pH and Redox Responsive Polymer Nanocompositesfor Cancer Therapy. J. Compos. Sci..

[30] Sun G., Wei X., Zhang D., Huang L., Liu H., Fang H. (2023). Immobilization of Enzyme Electrochemical Biosensors and Their Application to Food Bioprocess Monitoring. Biosensors.

[31] Eivazzadeh-Keihan R., Noruzi E.B., Chidar E., Jafari M., Davoodi F., Kashtiaray A., Gorab M.G., Hashemi S.M., Javanshir S., Cohan R.A. (2022). Applications of carbon-based conductive nanomaterials in biosensors. Chem. Eng. J..

[32] Kamalasekaran K., Magesh V., Atchudan R., Arya S., Sundramoorthy A.K. (2023). Development of Electrochemical Sensor Using Iron (III) Phthalocyanine/Gold Nanoparticle/Graphene Hybrid Film for Highly Selective Determination of Nicotine in HumanSalivary Samples. Biosensors.

[33] Mahesh M.S., Manasa G., Ronald J.M., Mondal K., Shetti N.P. (2023). Fundamentals of bio-electrochemical sensing. Chem. Eng. J. Adv..

[34] Mikuła E. (2021). Recent Advancements in Electrochemical Biosensors for Alzheimer’s Disease Biomarkers Detection. Curr. Med. Chem..

[35] Hwang H.S., Jae W.J., Yoong A.K., Mincheol C. (2020). Carbon Nanomaterials as Versatile Platforms for Biosensing Applications. Micromachines.

[36] Kim J., Park G., Lee S., Hwang S.W., Min N., Lee K.M. (2017). Single Wall Carbon Nanotube Electrode SystemCapable of Quantitative Detection of CD4^+^ T Cells. Biosens. Bioelectron..

[37] Wang L., Jia W., Wu Y., Wang F., Kui L. (2019). Direct Fabrication of 3D Graphene–Multi Walled Carbon Nanotubes Network and Its Application for Sensitive Electrochemical Determination of Hyperin. Int. J. Electrochem. Sci..

[38] Shantharaja, Giddaerappa, Sannegowda L.K. (2023). Phthalocyanine based metal-organic frame work with carbon nanoparticles as hybrid catalyst for oxygen reduction reaction. Electrochim. Acta.

[39] Leznoff C.C., Lever A. (1993). Phthalocyanines: Properties and Applications.

[40] Giddaerappa, Nemakal M., Shantharaja, Mirabbos H., Lokesh K.S. (2022). Tetraphenolphthalein Cobalt(II) Phthalocyanine Polymer Modified with Multiwalled Carbon Nanotubes as an Efficient Catalyst for the Oxygen Reduction Reaction. ACS Omega.

[41] Ozoemena K.I., Nyokong T. (2006). Novel amperometric glucose biosensor based on an ether-linked cobalt(II) phthalocyanine–cobalt(II) tetraphenylporphyrin pentamer as a redox mediator. Electrochim. Acta.

[42] Liu D., Long Y.T. (2015). Superior Catalytic Activity of Electrochemically Reduced Graphene Oxide Supported Iron Phthalocyanines toward Oxygen Reduction Reaction. ACS Appl. Mater. Interfaces.

[43] Pari M., Reddy K.R.V., Chandrakala K.B. (2020). Amperometric determination of dopamine based on an interface platform comprising tetra-substituted Zn^2+^ phthalocyanine film layer with embedment of reduced graphene oxide. Sens. Actuators A Phys..

[44] Sarvajith M.S., Harish M., Nimbegondi K., Mruthyunjayachari C.D., Fasiulla K. (2020). Synthesis and Electrochemical Investigation of Tetra Amino Cobalt (II) Phthalocyanine Functionalized Polyaniline Nanofiber for the Selective Detection of Dopamine. Electroanalysis.

[45] Veeresh A.S., Imadadulla M., Manjunatha N., Shambulinga A., Hemantha K.K.R., Swamy S., Lokesh K.S. (2019). Synthesis and electropolymerization of cobalt tetraaminebenzamidephthalocyanine macrocycle for the amperometric sensing of dopamine. J. Electroanal. Chem..

[46] Imadadulla M., Manjunatha N., Shambhulinga A., Veeresh A.S., Divakara T.R., Manjunatha P., Keshavanandaprabu C.P., Lokesh K.S. (2020). Phthalocyanine sheet polymer based amperometric sensor for the selective detection of 2,4-dichlorophenol. J. Electroanal. Chem..

[47] Manjunatha N., Giddaerappa, Shantharaja, Veeresh A.S., Lokesh K.S. (2021). Novel amide coupled phthalocyanines: Synthesis and structure-property relationship for electrocatalysis and sensing of hydroquinone. J. Electroanal. Chem..

[48] Sajjan V.A., Aralekallu S., Nemakal M., Palanna M., Prabhu C.K., Sannegowda L.K. (2020). Nanomolar detection of lead using electrochemical methods based on a T novel phthalocyanine. Inorganica Chim. Acta.

[49] Shantharaja, Nemakal M., Giddaerappa, Sannegowda L.K. (2021). Biocompatible polymeric pyrazolopyrimidinium cobalt(II) phthalocyanine: An efficient electroanalytical platform for the detection of l-arginine. Sens. Actuators A.

[50] Sajjan V.A., Aralekallu S., Nemakal M., Palanna M., Prabhu C.K., Sannegowda L.K. (2021). Nanomolar detection of 4-nitrophenol using Schiff-base phthalocyanine. Microchem. J..

[51] Keshavananda Prabhu C.P., Nemakal M., Aralekallu S., Mohammed I., Shivaprasad K.H., Amshumali M.K., Lokesh K.S. (2019). Synthesis and characterization of novel imine substituted phthalocyanine for sensing of L-cysteine. J. Electroanal. Chem..

[52] Gonçalo D., João C., Bruno V., Leticia G., Carina A., Maria A., João R., Pedro V.B. (2012). Noble Metal Nanoparticles for Biosensing Applications. Sensors.

[53] Liao Z., Yao L., Liu Y., Wu Y., Wang Y., Ning G. (2021). Progress on nanomaterials based-signal amplification strategies for the detection of zearalenone. Biosens. Bioelectron..

[54] Sumitha M.S., Xavier T.S. (2023). Recent advances in electrochemical biosensors—A brief review. Hybrid Adv..

[55] Yin D., Liu J., Bo X., Li M., Guo L. (2017). Porphyrinic metal-organic framework/macroporous carbon composites for electrocatalytic applications. Electrochim. Acta.

[56] Ullah S., Ur Rehman A., Najam T., Hossain I., Anjum S., Ali R., Shahid M.U., Ahmad Shah S.S., Nazir M.A. (2024). Advances in metal-organic framework@activated carbon (MOF@AC) composite materials: Synthesis, characteristics and applications. J. Ind. Eng. Chem..

[57] Channabasavana Hundi Puttaningaiah K.P., D Ramegowda S., Hur J. (2024). Polymer Tetrabenzimidazole Aluminum Phthalocyanine Complex with Carbon Nanotubes: A Promising Approach for Boosting Lithium-Ion Battery Anode Performance. ACS Appl. Energy Mater..

[58] Gonçalves J.M., Bernardo A.I., Paulo R.M., Lúcio A. (2021). Recent advances in electroanalytical drug detection by porphyrin/phthalocyanine macrocycles: Developments and future perspectives. Analyst.

[59] Nobuhle N., Sithi M., Tebello N. (2021). Electrocatalytic Activity of Cobalt Phthalocyanines Revisited: Effect of the Number of Oxygen Atoms and Conjugation to Carbon Nanomaterials. Electrocatalysis.

[60] Liu H., Guo K., Lv J., Gao Y., Duan C., Deng L., Zhu Z. (2017). A novel nitrite biosensor based on the direct electrochemistry of horseradish peroxidase immobilized on porous Co3O4 nanosheets and reduced graphene oxide composite modified electrode. Sens. Actuators B Chem..

[61] Bounegru A.V., Apetrei C. (2021). Development of a Novel Electrochemical Biosensor Based on Carbon Nanofibers–Cobalt Phthalocyanine–Laccase for the Detection of p-Coumaric Acid in Phytoproducts. Int. J. Mol. Sci..

[62] Nemakal M., Aralekallu S., Mohammed I., Swamy S., Sannegowda L.K. (2019). Electropolymerized octabenzimidazole phthalocyanine as an amperometric sensor for hydrazine. J. Electroanal. Chem..

[63] Jilani B.S., Mounesh, Malathesh P., Mruthyunjayachari C.D., Reddy K.V. (2020). Cobalt (II) tetra methyl-quinoline oxy bridged phthalocyanine carbon nano particles modified glassy carbon electrode for sensing nitrite: A voltammetric study. Mater. Chem. Phys..

[64] Shambhulinga A., Imadadulla M., Manjunatha N., Palanna M., Sannegowda L.K. (2019). Synthesis of novel azo group substituted polymeric phthalocyanine for amperometric sensing of nitrite. Sens. Actuators B Chemical..

[65] Manjunatha N., Shambhulinga A., Imadadulla M., Pari M., Reddy K.V., Sannegowda L.K. (2019). Nanomolar detection of 4-aminophenol using amperometric sensor based on a novel phthalocyanine. Electrochim. Acta.

[66] Manjunatha N., Shambhulinga A., Imadadulla M., Keshavananda Prabhu C.P., Lokesh K.S. (2018). Chemisorbed palladium phthalocyanine for simultaneous determination of biomolecules. Microchem. J..

[67] Reddy K.R.V. (2021). Decorated CoPc with appliance of MWCNTs on GCE: Sensitive and reliable electrochemical investigation of heavy metals. Microchem. J..

[68] Jilani B.S., Pari M., Reddy K.V., Lokesh K.S. (2019). Venugopala Reddy.; K.S. Lokesh. Simultaneous and sensitive detection of ascorbic acid in presence of dopamine using MWCNTs-decorated cobalt (II) phthalocyanine modified GCE. Microchem. J..

[69] Keshavanandaprabhu C.P., Manjunatha N., Shambhulinga A., Imadadulla M., Manjunatha P., Veeresh A.S., Akshitha D., Lokesh K.S. (2019). A comparative study of carboxylic acid and benzimidazole phthalocyanines and their surface modification for dopamine sensing. J. Electroanal. Chem..

[70] Mania V., Huanga S.T., Devasenathipathy R., Yang T.C.K. (2016). Electropolymerization of cobalt tetraamino-phthalocyanine at reduced graphene oxide for electrochemical determination of cysteine and hydrazine. RSC Adv..

[71] Tang C., Umasankar Y., Chen S.M. (2009). Simultaneous determination of adenine guanine and thymine at multi-walled carbon nanotubes incorporated with poly(new fuchsin) composite film. Anal. Chim. Acta.

[72] Keshavananda C.P., Kenkera R.N., Shambhulinga A., Shivalingayya, Lokesh K.S. (2023). Novel polymeric cobalt tetrabenzimidazole phthalocyanine for nanomolar detection of hydrogen peroxide. RSC Sustain..

[73] Imadadulla M., Manjunatha N., Veeresh A.S., Dayananda B.P., Lokesh K.S. (2018). Electropolymerized film of cobalt tetrabenzimidazolephthalocyanine for the T amperometric detection of H_2_O_2_. J. Electroanal. Chem..

[74] Gaddimath S., Chandrakala K.B., Lagashetty A., Dani S., Prabhu C.K., Giddaerappa, Sannegowda L.K. (2024). Ilmenite-type NiTiO_3_ nanoparticles for oxygen evolution reaction. J. Appl. Electrochem..

[75] Keshavananada Prabhu C.P., Aralekallu S., Sajjan V.A., Palanna M., Kumar S., Sannegowda L.K. (2021). Non-precious cobalt phthalocyanine-embedded iron ore electrocatalysts for hydrogen evolution reactions. Sustain. Energy Fuels.

